# Mild and Efficient One-Step Synthesis of Nitrogen-Doped Multistage Porous Carbon for High-Performance Supercapacitors

**DOI:** 10.3390/molecules28248136

**Published:** 2023-12-17

**Authors:** Tianliang Zhang, Jun Li

**Affiliations:** College of Chemical Engineering, Sichuan University, Chengdu 610065, China; zhangtianliang@stu.scu.edu.cn

**Keywords:** nitrogen-doped, porous carbon, solid K_2_CO_3_, supercapacitors

## Abstract

Biomass-derived carbon materials have broad application prospects in energy storage, but still face problems such as complex synthesis paths and the massive use of corrosive activators. In this study, we proposed a mild and efficient pathway to prepare nitrogen-doped porous carbon material (N-YAC) using one-step pyrolysis with solid K_2_CO_3_, tobacco straw, and melamine. The optimized material (N-YAC_0.5_) was not only enriched with nitrogen, but also exhibited a high specific surface area (2367 m^2^/g) and a reasonable pore size distribution (46.49% mesopores). When utilized in electrodes, N-YAC_0.5_ exhibited an excellent capacitance performance (338 F/g at 1 A/g) in the three-electrode system, and benefitted from a high mesopore distribution that maintained a capacitance of 85.2% (288 F/g) at high current densities (20 A/g). Furthermore, the composed symmetric capacitor achieved an energy density of 14.78 Wh/kg at a power density of 400 W/kg. In summary, our work provides a novel and eco-friendly approach for converting biomass into high-performance energy-storage materials.

## 1. Introduction

In recent years, with the persistent shortage of fossil fuels and growing pollution problems, countries worldwide have stepped up to develop and utilize clean energy, such as solar and wind power [1,2]. However, these energy sources are often intermittent and cannot provide continuous and stable output. Their utilization is inseparable from the support of advanced energy-storage equipment, which puts forward higher requirements for traditional energy-storage equipment. As an energy-storage device between batteries and traditional electrostatic capacitors, supercapacitors combine both advantages, featuring a long cycle life, a high power density, and fast charging and discharging [3,4,5]. They are expected to be the best candidates to meet the above requirements. However, the current supercapacitors still have a lower energy density, which reduces their range of applications. The performance of supercapacitors is largely dependent on the electrode materials, so enhancing the properties of electrode materials to achieve a high-performance output is necessary.

As is universally acknowledged, carbon-based materials are extensively employed as the electrode material in electric double-layer capacitors (EDLCs), owing to their cost-effectiveness, generally high specific surface area, and excellent chemical stability. Nanotextured carbon materials such as graphene [6,7], mesoporous carbon [8,9], carbon nanotubes [10,11], and carbon aerogel [12,13] have been shown to have excellent capacitive properties. However, intricate manufacturing methods and elevated costs hinder their applications. In recent years, some works on graphene-based materials′ low-cost and easily scalable synthesis have been reported [9,14,15], but their capacitive properties need to be enhanced. Commercial activated carbons are still the most common electrode materials today; unfortunately, they are mainly obtained from fossil fuels, and the precursors are environmentally unfriendly and expensive. Hence, using natural and abundant biowastes from domestic, industrial, animal, and municipal wastes to obtain carbon materials has attracted great attention [16,17,18]. Tobacco straw is the most dominant by-product of the tobacco industry, and the amount produced is about 4.5 × 10^6^ t per year in China [19]; most of it is discarded or incinerated, leading to environmental pollution and resource wastage. It is of excellent research significance to convert it into EDLC electrode materials for high-value utilization [20,21].

As EDLCs mainly rely on the charge–discharge process (physical sorption) in an electric double layer at the porous electrodes without an electrochemical reaction occurring [22], enhancing the SSA of biomass-carbon materials can effectively enhance their energy-storage capacity. Chemical activation is considered an efficient approach for increasing the porosity of biochar [23]. Zhao et al. introduced a tobacco-stem-derived hydrothermal carbon (TC) with KOH activation, which was optimized to achieve a maximum SSA and a specific capacitance of 2097 cm^2^/g and 286.6 F/g, respectively [24]. Another method to boost the energy-storage capacity of carbon materials is heteroatom doping (N, S, P), which improves the surface hydrophobicity of carbon and enhances the electronic conductivity and inductive pseudocapacitance [25]. Zou et al. found that the synergistic effect of KOH and urea modulated the pore size distribution of bagasse-based porous carbon and introduced a suitable nitrogen content, which resulted in an excellent capacitance performance (350.8 F/g) [26]. Yuan et al. prepared activated carbon (ATC) from tobacco straw using KOH activation and further doped it with thiourea to obtain an N and S co-doped carbon material (NS-ATC); the successful doping of nitrogen and sulfur increased the capacitance to 422.5 F/g [27]. However, these studies dealt only with KOH-activated systems, and there are few studies on doping heteroatoms in other activation modes. Carbon activated with KOH has a high SSA and a high porosity, but from an industrial point of view, the use of hydroxides for fabricating biomass-carbon materials is quite limited due to their highly corrosive properties at high temperatures. It is necessary to continue the search for greener, milder activators and to study the effect of heteroatom dopants under this activation.

In this work, novel N-doped porous carbon (N-YAC) was obtained in one step using mild solid K_2_CO_3_ as an activator, tobacco straw as a carbon source, and melamine as a dopant. The experiments did not require pre-carbonization or solvent impregnation, and the effects of the melamine introduction and dosage on the structure and properties of porous carbon were explored. According to the results, melamine not only played the role of a nitrogen source to introduce nitrogen-containing functional groups, but it also acted as a pore size regulator to increase the specific surface area and improve the mesopore ratio. The optimized porous carbon exhibited excellent electrochemical properties with both three and two electrodes.

## 2. Results

**Figure 1:**
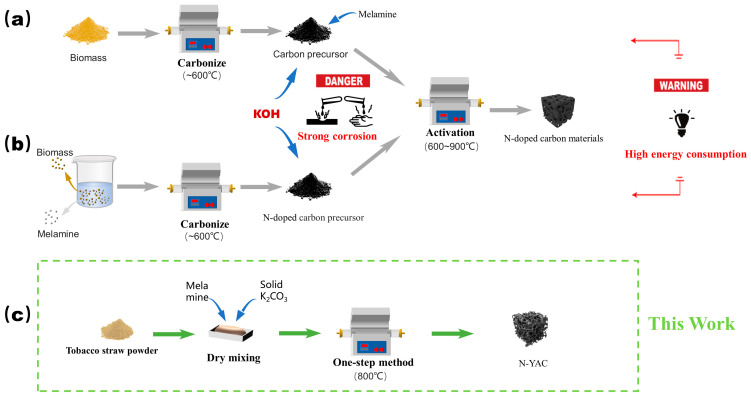
Comparison diagram for the preparation process of nitrogen-doped porous carbon: (**a**) carbonization followed by doping, (**b**) doping followed by carbonization, and (**c**) this work.

A comparison of this paper with the conventional method (using KOH as an activator) for synthesizing nitrogen-doped porous carbon is shown in Figure 1. The traditional synthesis routes are usually divided into two types: In one, the biomass is first carbonized at a low temperature to obtain the carbon precursor, and then the precursor is mixed with KOH and melamine and finally activated at a high temperature to obtain the nitrogen-doped samples (Figure 1a) [28,29,30]. In the other, as shown in Figure 1b, the biomass is first impregnated with the melamine solution, and then sequentially carbonized and activated to obtain the nitrogen-doped samples [31,32]. In this study (Figure 1c), the crushed tobacco straw was first dry-mixed with melamine and solid K_2_CO_3_, and then carbonized, activated, and doped in one step at 800 °C to obtain the product. Compared with the synthetic route using KOH as the activator, this method did not require impregnation or step-by-step carbonization activation. This simplified the process, reduced the energy input, lowered the production cost, and was more economical and environmentally friendly. In addition, since the chemical nature of K_2_CO_3_ is mild, it requires less corrosion protection for the reaction equipment and is easier to promote industrially.

**Figure 2:**
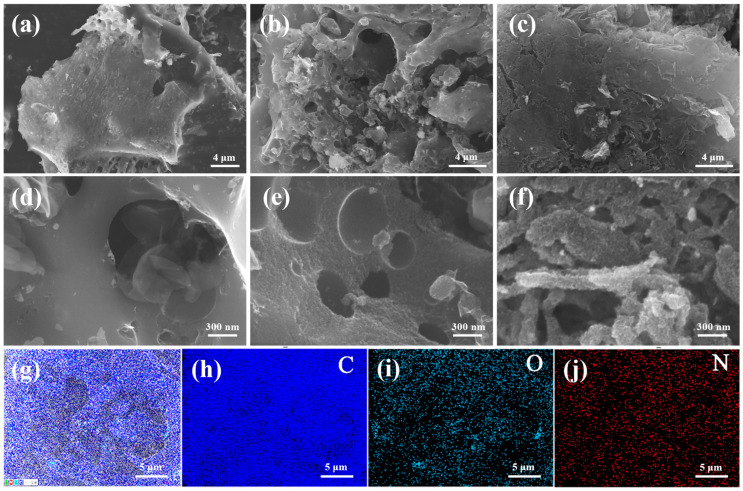
SEM images for (**a**,**d**) YAC, (**b**,**e**) N-YAC_0.5_, and (**c**,**f**) N-YAC_2_. (**g**) EDS-element-layered image and elemental mapping image of (**h**) C, (**i**) O, and (**j**) N for N-YAC_0.5_.

The morphology of the so-prepared samples was characterized using SEM. As presented in Figure 2, the YAC surface was relatively flat with a few wrinkled structures, and some pore structures were observed at the edges. It was formed by the reduction of K_2_CO_3_ by carbon in an inert atmosphere (K_2_CO_3_ + 2C → 2K + 3CO), which is known as the activation process. With the introduction of melamine, the surface of N-YAC_0.5_ exhibited many large circular pore structures, which usually means that a large amount of internal gas escaped from the sample surface. The high-magnification image (Figure 2d) shows that, compared with YAC, the pore walls of N-YAC_0.5_ became roughened, and uniform interconnected micropores/mesopores appeared. These interconnected pore structures can improve ion transfer efficiency and enhance the electrochemical performance [33]. As the ratio of melamine to tobacco straw rose to 2:1, the overall structure of N-YAC_2_ was disrupted into smaller fragments and piled up, with the surface and pore walls becoming rougher. The structural damage may have affected channel intercommunication, causing a reduction in the ion transport capacity. The mapping image of N-YAC_0.5_ shows that the O and N elements were uniformly scattered throughout the entire carbon skeleton.

**Figure 3:**
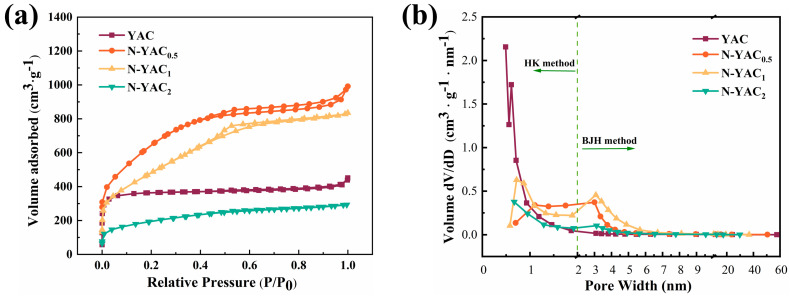
(**a**) N_2_ adsorption–desorption isotherms of YAC and N-YACx and (**b**) pore size distribution.

The SSA and pore size distributions for YAC and N-YACx were calculated using N_2_ adsorption–desorption isotherms, and the isotherm curves of the prepared carbon materials in Figure 3a conformed to the type I/IV hybrid isotherms (IUPAC classification). When the relative pressures were lower than 0.05, the adsorbed amount of N_2_ increased dramatically with increasing relative pressure, which was caused by the microporous filling effect, confirming the occurrence of many microporous structures in the samples [34]. The H4-type hysteresis loops at higher relative pressures (above 0.5) were related to capillary condensation, illustrating the presence of a microporous/mesoporous structure. The hysteresis loop was more pronounced for N-YACx than for YAC, indicating that mesopores may have been more abundant after the introduction of melamine. The pore size distributions corresponding to the isotherm were calculated using the HK and BJH models. Figure 3b shows that the pore size distribution of YAC was relatively narrow and concentrated around 0.5 nm. In contrast, N-YACx had a broader pore size distribution, ranging from 0.5 to 1.5 nm and 2 to 6 nm. Micropores (<2 nm) can capture electrolyte ions and store charge, while mesopores (2–50 nm) can offer appropriate channels for electrolyte permeation and ion transport.

The SSA and pore volume data of YAC and N-YACx are tallied in Table 1. With the introduction of melamine, the SSA, V_micro_, and V_meso_ of N-YAC_0.5_ increased significantly, and V_meso_/V_total_ increased from 21.95% to 46.49%. This indicates that melamine promoted the formation of more new micropores into the carbon skeleton and contributed to the expansion of micropores to form mesopores, which increased the SSA of the samples [28]. The sudden reduced SSA and V_total_ exhibited by N-YAC_2_ indicates that an overdose of melamine will cause excessive ablation and pore structure collapse, which is consistent with the SEM observations. In conclusion, introducing melamine and adjusting the dosage can allow for the preparation of a hierarchical, structured, porous carbon with a higher SSA.

**Figure 4:**
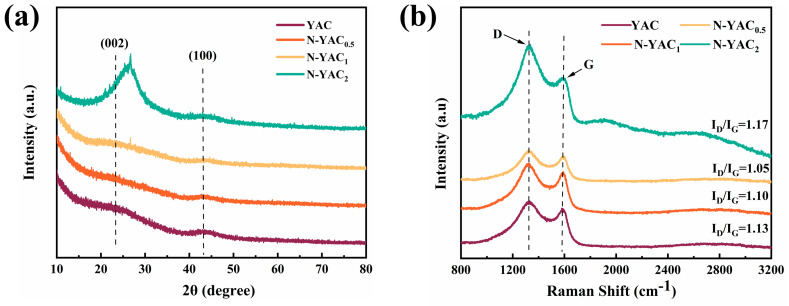
(**a**) XRD patterns of YAC and N-YACx (x = 0.5, 1, 2); (**b**) Raman spectrum of YAC and N-YACx (x = 0.5, 1, 2).

The XRD and Raman energy spectrums were analyzed to better determine the materials’ crystalline shape, phase state, and degree of graphitization. As can be observed from Figure 4a, two broad humps around 23° and 44° were present in all the XRD profiles, representing the (002) and (100) crystal planes of the graphite crystals, sequentially [35]. This demonstrates that the prepared samples were typical amorphous carbon with small numbers of graphite structures. In addition, compared to the other samples, the peak of N-YAC_2′_s (002) was more visible and shifted to a higher angle. The d_002_ pitch of N-YAC_2_ was calculated as 0.340 nm, which is very close to that of intact graphite (0.335 nm), demonstrating that N-PAC_2_ holds a higher degree of crystallinity [36]. These carbon structure variations can be more clearly observed using Raman spectroscopy. Figure 4b shows that two drum peaks occurred at 1354 cm^−1^ and 1590 cm^−1^, corresponding to peaks D (disordered peak) and G (graphite peak), respectively. It is generally considered that the ratio of the intensity of the two peaks (I_D_/I_G_) is minor when the graphitization level of the measured carbon material is higher [37]. In this study, the I_D_/I_G_ values of YAC, N-YAC_0.5_, and N-YAC_1_ were 1.13, 1.10, and 1.05, respectively. The results illustrate that the degree of graphitization gradually increased as the amount of melamine increased, which indicates that melamine interacted with the tobacco straw during pyrolysis and promoted graphite structure generation. Notably, the D peak of N-YAC_2_ was abnormally enhanced, as its I_D_/I_G_ increased to 1.17, which implies that high-ratio melamine doping led to a more disordered carbon material. This was attributed to the decomposition and substitution of the high-ratio nitrogen dopant, which introduced more defects (vacancies and topological defects) in the carbon structure, disrupting the spin and electronic order of the carbon units [38,39].

**Figure 5:**
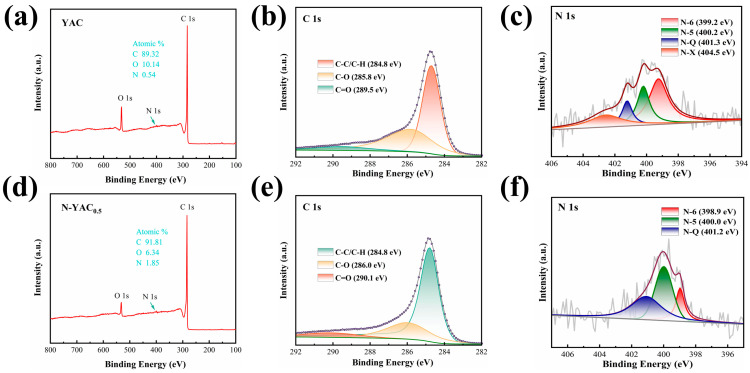
XPS survey spectra for (**a**) YAC and (**b**) N-YAC_0.5_; high-resolution C 1s spectra for (**c**) YAC and (**d**) N-YAC_0.5_; and high-resolution N 1s spectra for (**e**) YAC and (**f**) N-YAC_0.5_.

The mode of occurrence of the elements on the material′s surface was analyzed using XPS. Figure 5a,b show that carbon was the primary element in the sample, in addition to small amounts of nitrogen and oxygen. The intensity of the O 1s peak of N-YAC_0.5_ was attenuated compared with that of YAC, indicating that the reaction of tobacco straw with melamine removed part of the oxygen element in the carbon material, which may be beneficial for increasing the conductivity and Coulombic efficiency of the material. The total peaks in the N 1s spectrum of YAC can be deconvoluted into four individual N peak components: pyridinic-N (N-6, 399.2 eV), pyrrolic-N (N-5, 400.2 eV), quaternary-N (N-Q, 401.3 eV), and nitrogen–oxygen (N-X, 404.5 eV). In comparison, the N 1s spectrum of N-YAC_0.5_ can be deconvoluted into three individual N peak components: N-6 (398.9 eV), N-5 (400.0 eV), and N-Q (401.2 eV). The relative positions of nitrogen among the various chemical states in the carbon skeleton are shown in Figure S1.

Table 2 compares the changes in nitrogen of the samples before and after melamine incorporation. The YAC without doping contained fewer nitrogen elements and mainly consisted of N-6 (44%). While the total nitrogen content of N-PAC_0.5_ increased from 0.54 at% to 1.85 at% after doping, the percentage of N-6 decreased and was mainly composed of N-5 (42%) and N-Q (40%). Generally, N-5 and N-6 in carbon are considered to be electroactive sites that contribute to the generation of pseudocapacitance and increase the electric capacity, whereas positively charged N-X and N-Q can promote the transfer of electrons through carbon and improve the capacitance at high current loads [40]. The introduction of melamine increased the nitrogen content in the carbon material and changed its chemical state.

**Figure 6:**
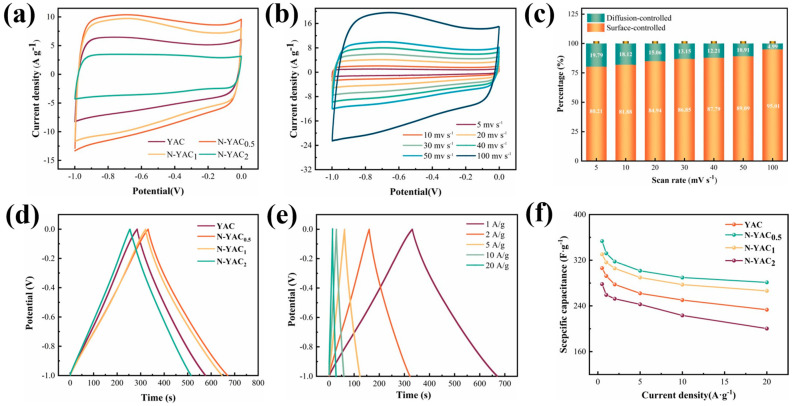
(**a**) Comparison of CV curves at 50 mv/s; (**b**) CV curves of N-PAC_0.5_; (**c**) capacitance contribution of N-PAC_0.5_ at different scanning rates; (**d**) comparison of CV curves at 1 A/g; (**e**) GCD curves of N-PAC_0.5_; and (**f**) specific capacitance of different samples.

The electrochemical performances of YAC and N-YACx were evaluated in a 6 M KOH electrolyte with a three-electrode system. As can be seen from Figure 6a, the CV curves for YAC and N-YAC_x_ were approximately rectangular at 50 mV/s. This indicates that the samples had low charge impedance and excellent ion transport properties, and the introduction of melamine did not alter their energy-storage mechanism. At the same scan rate, the electrodes′ capacitance was positively linked to the integral area of their CV curves. Evidently, the capacitance of N-YAC_0.5_ was higher than in other samples. Figure 6b presents the performance of N-YAC_0.5_ at a broader range of scan rates, with the CV curves consistently conforming to the rectangular shape, indicating an excellent potential stability.

The charge storage mechanism for N-YAC_0.5_ can be obtained by calculating the logarithmic relationship between the scan rate (v) and the peak current (i), expressed as *logi* = *blogv* + *loga*. The intercept b = 1 implies that the surface control process determines the charge storage and exhibits capacitive behavior. When b = 0.5, it reveals a mechanism dominated by diffusion-controlled processes, which corresponds to the battery characteristics. Figure S2 shows that the b values for the charging and discharging of the fitted N-YAC_0.5_ were 0.95 and 0.91, respectively, indicating that capacitive surface processes dominated its storage mechanism. Dunn′s formula can further quantify the contributions of the surface-controlled and diffusion-controlled storage mechanisms [41]:

(1) $$i(v)=k_{1}v+k_{2}v^{0.5}$$

where *k*_2_*v*^0.5^ and *k*_1_*v* represent the capacitance contribution of the slow kinetic process (diffusion control) and the fast kinetic process (surface control), respectively. As shown in Figure 6c, even at a low sweep speed (5 mv/s), the surface process still contributed 80.21% of the capacitance, which was attributed to the high mesopore ratio of N-YAC_0.5_, ensuring fast ion transport in the channel.

The GCD curves of YAC and N-YACx at 1 A/g are given in Figure 6d. All the curves in the figure followed symmetric triangles, indicating that the EDLCs of the samples had desirable capacitive behavior. The capacitances calculated from the GCD curves of the four samples according to Equation (2) are shown in Figure 6f. The specific capacitance of N-YAC_0.5_ at 1 A/g was 338 F/g and still retained 82% of the capacitance even when the current density was increased to 20 A/g, which was higher than that of YAC (288 F/g and 73%). This demonstrates that introducing melamine is vital for boosting the samples′ specific capacitance and rate performance. From Table 3, it can be clearly seen that the capacitance of N-YAC_0.5_ was significantly improved over the previously reported work and was even superior to some of the KOH-activated products, suggesting that the use of melamine-assisted K_2_CO_3_ activation to produce nitrogen-doped porous carbons holds excellent promise.

**Figure 7:**
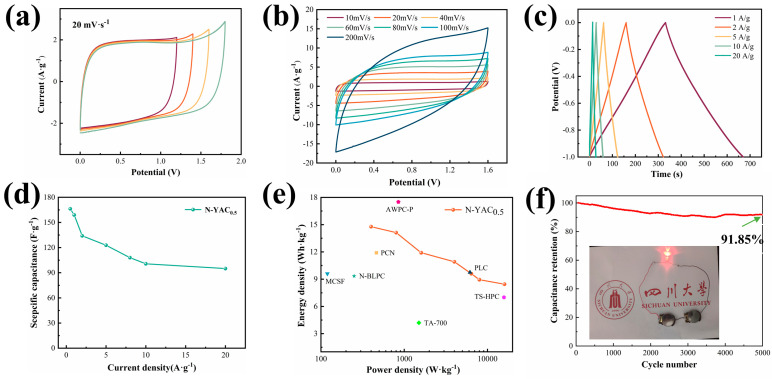
Electrochemical performance of symmetrical N-YAC_0.5_//N-YAC_0.5_ capacitors: (**a**) CV curves at different potential windows; (**b**) CV curves at 10–200 mv/s; (**c**) GCD curves at 0.5–20 A/g; (**d**) capacitance versus current density; (**e**) Ragone plots; and (**f**) continual galvanostatic charge for 5000 cycles, tested at current density of 1.0 A/g.

The potential of N-YAC_0.5_ for practical applications was investigated by assembling two equal-mass N-YAC_0.5_ electrodes into a symmetrical capacitor using 1 M Na_2_SO_4_ as the electrolyte. As shown in Figure 7a, the CV curves of the supercapacitor were gradually polarized with an increase in the window voltage, and this was more evident after exceeding 1.6 V, which was due to the occurrence of HER/OER in the aqueous electrolyte [46]. Figure 7b shows that the CV curves of N-YAC_0.5_ at 5–200 mv/s were all approximately rectangular, indicating that N-YAC_0.5_ had a highly reversible capacitive performance. Figure 7c summarizes the GCD curves of the capacitor at 0.5–20 A/g. The curves showed a symmetrical triangular shape, and the N-YAC_0.5_ had a high Coulomb efficiency (~97.44%). The calculated capacitance of N-YAC_0.5_ at 0.5 A/g was 166.25 F/g, at which point the energy density reached a maximum of 14.78 Wh/kg. The capacitor maintained an energy density of 8.44 Wh/kg, even at a high power density of 16,000 W/kg, which is competitive with previous reports [8,21,29,42,44,47]. In addition, cycle stability is an important metric for supercapacitor applications. It examines the capacity retention of the active material after a certain number of rapid charges and discharges and is essential for evaluating the lifetime of supercapacitors. As shown in Figure 7f, the N-YAC_0.5_ electrode maintained a specific capacity of 91.85% after 5000 cycles (1 A/g), demonstrating its excellent cycling stability. This was attributed to the fact that nitrogen doping reduces the oxygen content in N-YAC0.5 and creates a relatively high pore volume and a robust pore system network, which improves the chemical properties and porous structure stability. Finally, the 2 V LED beads were successfully lit by two series-connected symmetrical N-YAC_0.5_ capacitors.

**Figure 8:**
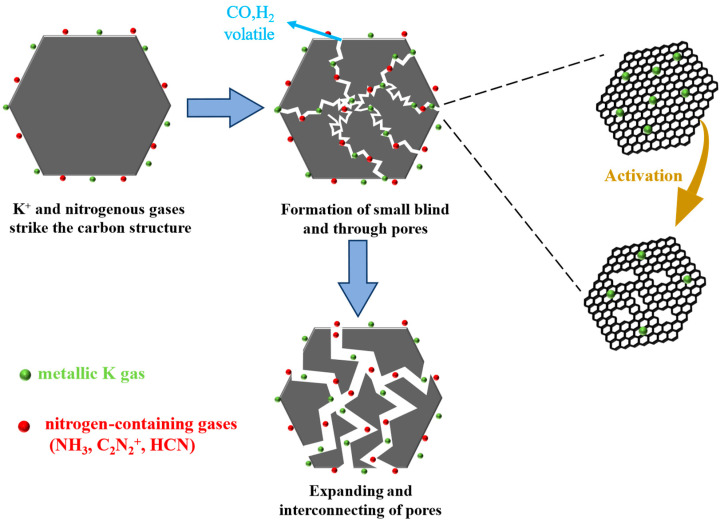
Schematic diagram of pore formation under the synergistic effect of K_2_CO_3_ and melamine.

To investigate the principle of synergism between melamine and K_2_CO_3_, the weight loss behavior of tobacco straw and melamine was determined under a nitrogen atmosphere. As can be seen from Figure S3, the weight loss of tobacco straw could be divided into three parts. The first stage occurred at 80–120 °C, with a low weight loss (4%), corresponding to the removal of water and the volatilization of a small number of light components. The second stage was in the 230–350 °C temperature range, which was tobacco straw′s leading weight loss interval and was related to the pyrolysis of cellulose, hemicellulose, and some lignin. The high temperature broke their chemical bonds and produced many volatile substances, including non-condensable small molecules such as H_2_, CO, CO_2_, and high-boiling hydrocarbons that can be condensed into a liquid form [48]. The third stage of weight loss in tobacco straw occurred after 500 °C, as the slow decomposition of the remaining lignin and the secondary decomposition of tar. The weight loss interval of melamine is 230–350 °C, and melamine decomposes almost entirely, generating gaseous products such as HCN, NH_3_, and C_2_N_2_^+^ [30]. It is not difficult to see that the pyrolysis region of melamine overlaps with the main pyrolysis region of tobacco straw. When mixed and heated, the initial decomposed tobacco straw generated some free radicals, which combined with melamine and accelerated its decomposition. However, this mutual promotion only occurred on the surface of the tobacco straw particles, and it was not easy to involve the interior, which did not contribute much to the specific surface area [49]. When K_2_CO_3_ was added to the system, the activation effect (Equations (S1)–(S3)) made many tiny pores appear on the surface of the particles. The interaction between melamine and tobacco straw was able to extend to the interior of the particles, which prompted the micropores produced by activation to expand and form mesopores and macropores. This is consistent with the phenomena observed using SEM and BET. In addition, the biomass lowered the melting point of K_2_CO_3_, which could be partially converted to a molten state below 800 °C [50]. The molten K_2_CO_3_ encapsulated the tobacco straw and melamine, which inhibited the escape of the gaseous products of the two, allowing these reactions to proceed adequately. As shown in Figure 8, the excellent performance of N-YAC_0.5_ was due to the synergistic effect of the tobacco straw, melamine, and K_2_CO_3_.

## 3. Materials and Methods

### 3.1. Materials

Waste tobacco straw was supplied by the Sichuan Tobacco Quality Supervision and Test Station (Chengdu, China). It was pulverized to 100 mesh using a home powder grinder and then dried for 12 h at 80 °C. Potassium carbonate (K_2_CO_3_) and hydrochloric acid (HCl) were procured from Jinshan Chemical Co., Ltd. (Chengdu, China). Melamine (C_3_H_6_N_6_) was purchased from Kelong Chemical Co., Ltd. (Chengdu, China). All reagents were used untreated.

### 3.2. Preparation of N-Doped Porous Carbon

The tobacco straw powder, melamine, and solid K_2_CO_3_ were added proportionally to the mortar (1:x:2) and then sufficiently ground to form a homogeneous mixture. After that, the mixture was transferred to a corundum container with a lid, heated at 800 °C for 100 min in a tube at a ramp rate of 10 °C/min under argon protection, and then naturally cooled. The collected sample was added to a beaker with 1 M HCl and stirred for 30 min to remove the excess activator and other by-products, such as inorganic salts. Finally, the filtered product was washed thoroughly with distilled water and dried at 120 °C for 12 h. The prepared product was noted as N-YACx, where x denotes the mass ratio of tobacco straw powder to melamine (x = 0.5, 2, 2). In addition, as a comparison, the product prepared without the addition of melamine was named YAC.

### 3.3. Characterization

An SU3500 scanning electron microscope (Thermo, Waltham, MA, USA) was utilized to determine the morphology and size of the samples. The crystalline phases of the samples were obtained using an X Pert Pro MPD X-ray diffractometer (PANalytical, Almelo, The Netherlands) at 10°–80° (CuKα, 1.54 Å) and a LabRAM Odyssey Raman spectrometer (HORIBA, Kyoto, Japan) with a laser wavelength of 532 nm. X-ray photoelectron spectroscopy (XPS) was performed using an AXIS Supra (Kratos, Kyoto, Japan) equipped with an Al Ka X-ray source and used to analyze the surface chemistry of the samples. Nitrogen adsorption isotherm data were obtained with a QUADRASORB SI specific-surface-area-and-pore-size analyzer (Anton Paar, Graz, Austria), and the specific surface area (SSA) was calculated with a BET model. The pyrolysis process for each component was determined using a TGA/DSC 2 system (Mettler Toledo, Zurich, Switzerland).

All electrochemical measurements were conducted using a CS2350H workstation (Corrtest, Wuhan, China) in a three-electrode system. In this system, 6 M KOH was used as the electrolyte, with Hg/HgO and 10 × 10 mm platinum sheets as the reference and counter electrodes, respectively. For the preparation of the working electrode, the active material (YAC or N-YACx), the binder polytetrafluoroethylene (PTFE), and conductive carbon black (80:10:10 ratio) were homogeneously mixed in anhydrous ethanol to form a slurry, and then coated onto a nickel foam grid (1 cm × 1 cm). After vacuum-drying at 120 °C for 6 h, the nickel foam was pressed at 10 MPa to obtain the working electrode. The mass of active material on each working electrode was about 4 mg. A symmetric supercapacitor can be constructed using two carbon electrodes of equal mass in the aqueous electrolyte of 1 M Na_2_SO_4_.

In the three-electrode system, following the discharge process from the GCD curve, the capacitance per unit mass can be calculated using Equation (2):

(2) $$C_{g}=\frac{I\times \Delta t}{m\times \Delta V}$$

where *C_g_* (F/g), *I* (A), and *m* (g) represent the specific capacitance of the active material, the constant potential during the discharge, and the mass of the active material on the working electrode, respectively. Δ*t* (s) represents the time taken by the discharging process and Δ*V* is the range of potentials during discharge.

In the symmetric supercapacitor system assembled using CR2032, the calculation of the specific capacitance (F/g) (a single electrode) is based on Equation (3):

(3) $$C_{s}=\frac{4I\times \Delta t}{m\times \Delta V}$$

Unlike Equation (1), here, *m* (g) represents the combined mass of the active material on both electrodes [33].

The energy density (*E*, Wh/ kg) and power density (*P*, W/kg) of the whole system were calculated using Equations (4) and (5), respectively [51]:

(4) $$E=\frac{C_{s}\times \Delta V^{2}}{2\times 4\times 3.6}$$

(5) $$P=\frac{E_{t}}{\Delta t}\times 3600$$

All the CV and GCD tests were cycled for seven cycles, and the data from the fifth cycle were taken for further graphing and analysis.

## 4. Conclusions

Nitrogen-doped porous carbon (N-YAC) was prepared in a one-step pyrolysis by co-pyrolyzing a biomass carbon source (tobacco straw) with a nitrogen source (melamine) using mild K_2_CO_3_ as an activator. Melamine played an important role in forming the porous carbon, which optimized the nitrogen-doping level and modified the pore structure synergistically with K_2_CO_3_. The optimized N-PAC_0.5_ exhibited a high SSA (2367.40 m^2^/g) and a hierarchical porous structure (D_p_ = 2.60 nm, V_meso_/V_total_ = 46.49%). As a result of these advantages, N-PAC_0.5_ provided a maximum capacitance of 338 F/g in three electrodes (at 1 A/g) and an excellent rate capability with 82% capacitance at 20 A/g. More importantly, the symmetric capacitor assembled using N-PAC_0.5_ in a 1 M Na_2_SO_4_ electrolyte exhibited a desirable energy density (14.78 Wh/kg at 400 W/kg) and a long cycle stability (91.85% after 5000 cycles of charge/discharge). These results indicate that N-PAC_0.5_ is a promising electrode material. The one-step pyrolysis of biomass using solid K_2_CO_3_ and melamine is a green, efficient, and promising strategy to prepare nitrogen-doped porous carbon for high-performance electrodes.

## Figures and Tables

**Table 1:** Surface area and pore volume data for YAC and N-YACx.

Samples	S_BET_ (m^2^·g^−1^)	V_total_ (cm^3^·g^−1^)	V_meso_ (cm^3^·g^−1^)	V_micro_ (cm^3^·g^−1^)	V_meso_/V_total_ (%)	D_p_ (nm)
YAC	1110	0.70	0.15	0.56	21.95	2.52
N-YAC_0.5_	2367	1.53	0.71	0.98	46.49	2.59
N-YAC_1_	1773	1.29	0.85	0.73	65.72	2.91
N-YAC_2_	670	0.45	0.21	0.30	47.05	2.68

**Table 2:** Nitrogen content in samples.

Sample	Total Nitrogen (at. %)	Percentage of Components (%)			
		N-6	N-5	N-Q	N-X
YAC	0.54	44	24	12	20
N-YAC_0.5_	1.85	18	42	40	-

**Table 3:** Electrode properties of some biomass carbon materials reported in the past.

Materials	Activation Method	SSA (m^2^·g^−1^)	Electrolyte	Capacitance (F·g^−1^)	Ref.
Pine nutshell	KOH + melamine	1847	6 M KOH	324 (at 0.05 A·g^−1^)	[29]
Houttuynia	KOH + melamine	2090	6 M KOH	473.5 (at 1 A·g^−1^)	[28]
Reed straw	KOH + melamine	547.1	6 M KOH	202.8 (at 1 A·g^−1^)	[37]
Rice straw	KOH + melamine	2646	6 M KOH	337 (at 0.5 A·g^−1^)	[42]
Bagasse via	KOH + urea	2905.4	2 M Li_2_SO_4_	259.5 (at 1 A·g^−1^)	[26]
Miscanthus	KOH	639	6 M KOH	162 (at 0.5 A·g^−1^)	[43]
Kombucha	KOH	917	6 M KOH	326 (at 1 A·g^−1^)	[44]
Mangosteen Shell	K_2_CO_3_	2802.6	6 M KOH	298.2 (at 0.5 A·g^−1^)	[45]
Tobacco straw	K_2_CO_3_ + melamine	2367	6 M KOH	338 (at 1 A·g^−1^)	This work

## Data Availability

Data is contained within the article or supplementary material.

## Supplementary Materials

The following supporting information can be downloaded at: https://www.mdpi.com/article/10.3390/molecules28248136/s1, Figure S1: The mode of existence of nitrogen atoms in the carbon skeleton; Figure S2: Plot of fitted b value; Figure S3: TG and DTG curves: (a) melamine; (b) tobacco straw; and (c) melamine:tobacco straw = 1:1.
