# Supplementary material for: Mild and Efficient One-Step Synthesis of Nitrogen-Doped Multistage Porous Carbon for High-Performance Supercapacitors
Source: Molecules. 2023 Dec 17;28(24):8136. doi: 10.3390/molecules28248136 (PMC10745835; doi:10.3390/molecules28248136)
Supplement: Supplementary file 1 [file molecules-28-08136-s001.zip › molecules-2750841-supplementary.pdf]

Supplementary materials

Mild and efficient One-step synthesis of nitrogen-doped multistage porous carbon for high-performance supercapacitors

Tianliang Zhang [1] and Jun Li [1,] *

[1] College of Chemical Engineering, Sichuan University, Chengdu 610065, China

* Correspondence: lijun@scu.edu.cn

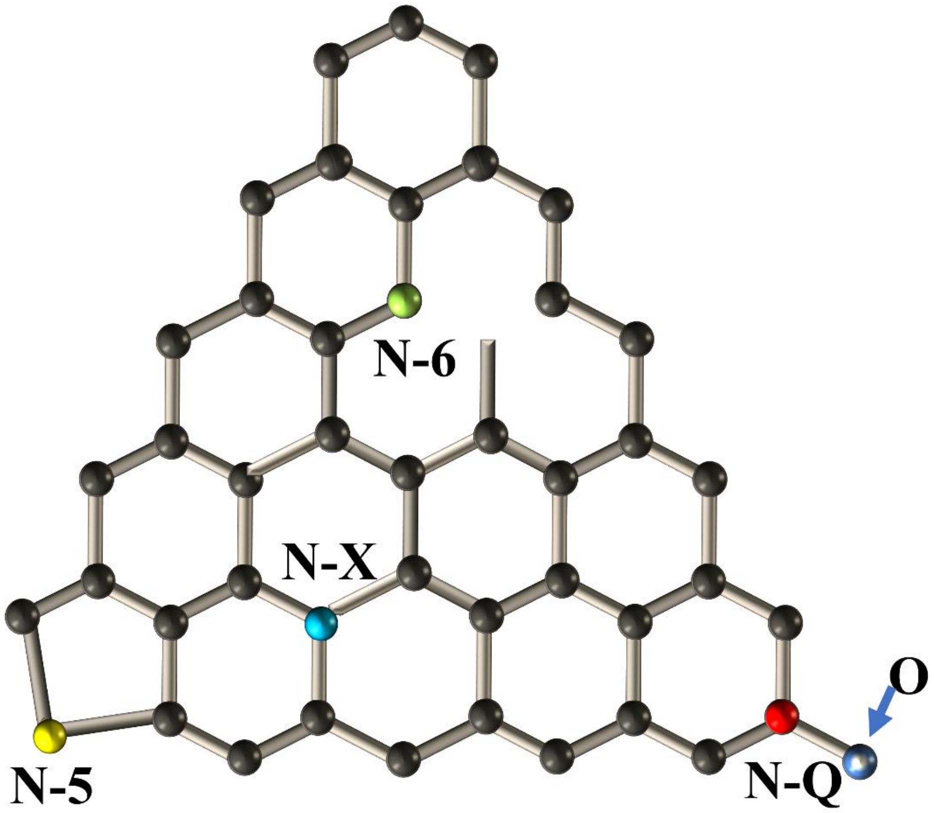

Figure S1. The mode of existence of nitrogen atoms in the carbon skeleton

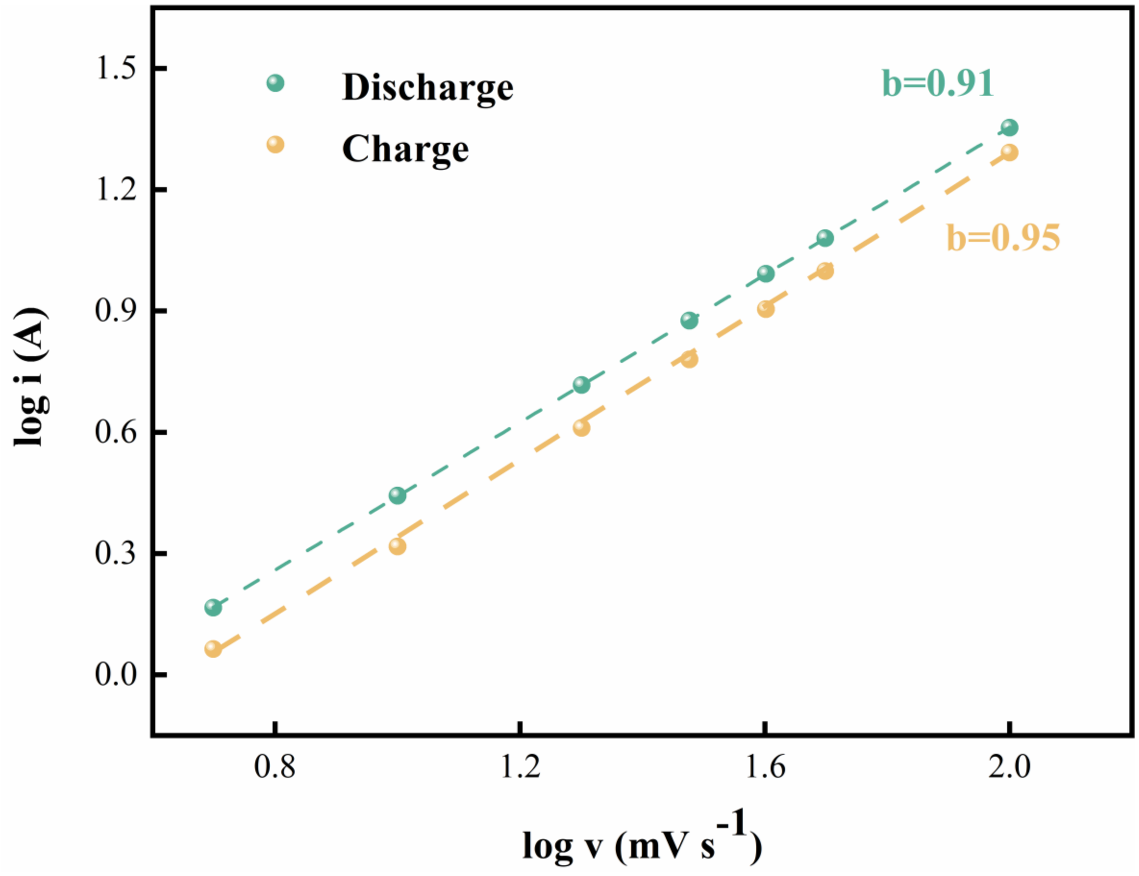

Figure S2. Fitted plots of peak current versus scan rate for the charging process (orange curve) and the discharging process (green curve) for N-PAC$_{0.5}$, fitted slopes are the values of b.

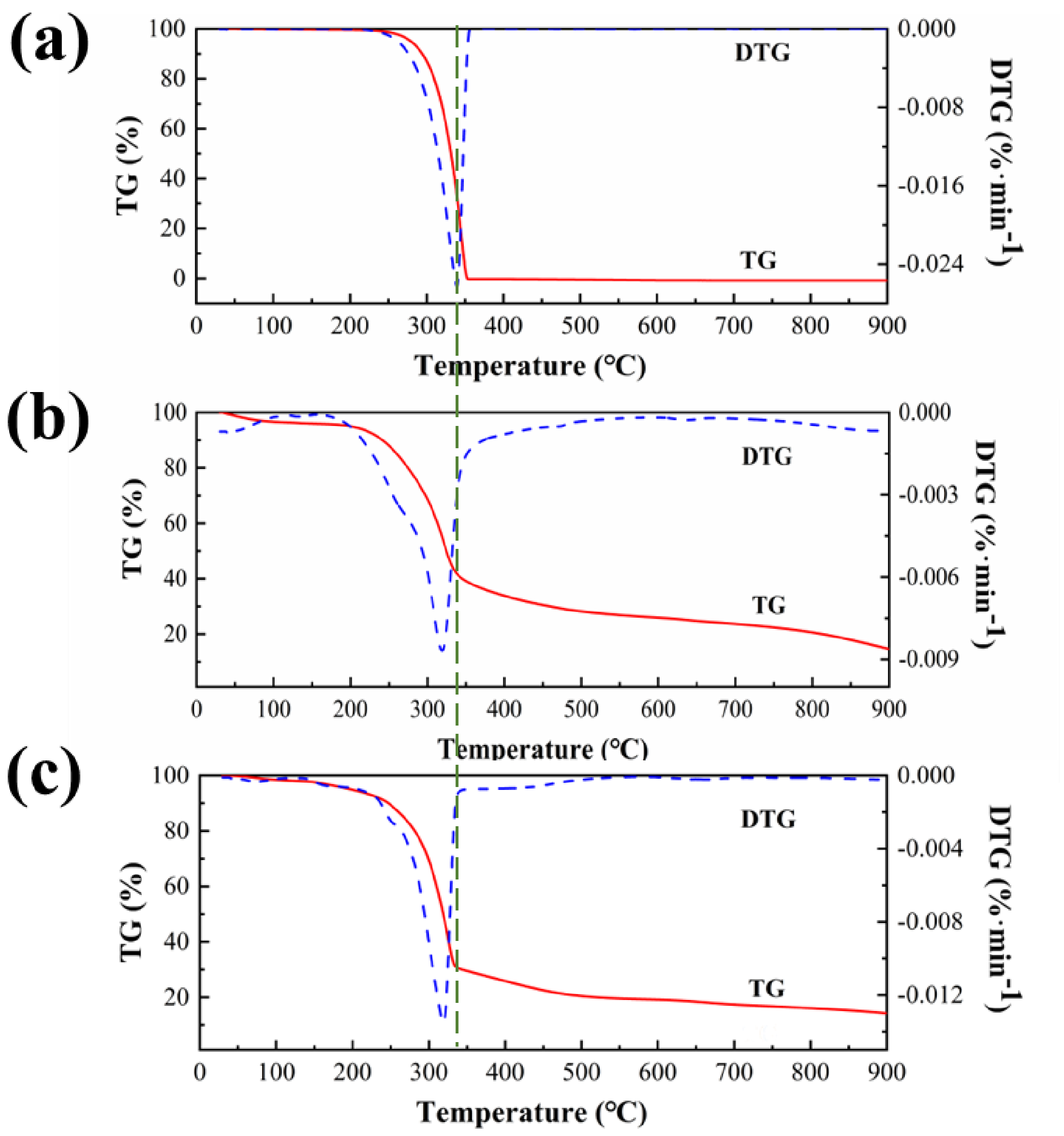

Figure S3. TG and DTG curves: (a) melamine; (b) tobacco straw;(c) melamine: tobacco straw =1:1

Detailed information on K_2CO_3 activation

During the activation process, K_2CO_3 decomposes into CO_2 and K_2O, which reacts with C atoms and then generates K and CO. When the temperature exceeds the boiling point of K (about 800 °C), the K vapor drills into the carbon layer and causes damage to the structure of the carbon material. In addition, the CO_2 produced during the activation process also plays a physical porosity role on the carbon structure [40].

$$K_2CO_3 \rightarrow K_2O + CO_2 \quad \text{(S1)}$$

$$K_2CO_3 + 2C \rightarrow 2K + 3CO \quad \text{(S2)}$$

$$K_2O + C \rightarrow 2K + CO \quad \text{(S3)}$$
